# Digital Leadership Scale for Clinical Nurses: Development and Validation of an Instrument

**DOI:** 10.2196/82101

**Published:** 2026-06-22

**Authors:** Ji-Hyeon Lee, Sun-Young Jung

**Affiliations:** 1 Department of Nursing, Yeungjin University, Daegu, Republic of Korea; 2 Research Institute of Nursing Science, College of Nursing, Daegu Catholic University, 33, Duryugongwon-ro 17-gil, Nam-gu, Daegu, 42472, Republic of Korea, 82 53-650-4977, 82 53-650-4392

**Keywords:** nurses, digital technology, leadership, digital health, factor analysis, statistical, nursing informatics, surveys and questionnaires

## Abstract

**Background:**

The rapid advancement of digital technologies, combined with the evolving complexity of health care environments, has introduced a new paradigm in nursing practice. Clinical nurses are now required not only to deliver safe and effective patient care but also to demonstrate competencies in digital literacy and innovation. Among these emerging competencies, digital leadership has become a critical attribute—enabling nurses to lead digital transformation, ensure patient safety, enhance care quality, and support system-level change within health care organizations. Despite its increasing relevance, there is a notable absence of validated measurement tools tailored to assess digital leadership in clinical practice.

**Objective:**

This study aimed to develop and psychometrically validate a Digital Leadership Scale for Clinical Nurses (DLS-CN) to systematically evaluate the digital leadership capabilities of nurses working in clinical settings.

**Methods:**

The scale development process followed a rigorous multistep procedure. Initial items were derived from previous qualitative research involving a literature review and in-depth interviews, complemented by an additional literature review conducted in this study. The content validity of 38 preliminary items was evaluated by 9 experts over 2 rounds. A pilot test was conducted with 30 nurses, followed by cognitive interviews with 5 nurses to refine item clarity and relevance. The final set of items was administered to 446 clinical nurses across various health care institutions. Data were randomly split for exploratory factor analysis and confirmatory factor analysis. Additional analyses were conducted to evaluate item discrimination, convergent validity, and internal consistency using IBM SPSS 25.0 and AMOS 23.0.

**Results:**

The finalized DLS-CN consists of 29 items grouped under four domains: (1) ability to use digital technology, (2) digital safety management, (3) digital collaboration mindset, and (4) organizational influence. These 4 factors explained 56.9% of the total variance. The scale showed strong internal consistency (Cronbach α=0.95). Convergent validity was demonstrated through strong positive correlations with the Nursing Informatics Competency Scale (Pearson correlation coefficient *r*=0.82; *P*<.001) and the Self-Leadership Scale (Pearson correlation coefficient *r*=0.83; *P*<.001).

**Conclusions:**

The DLS-CN is a valid and reliable instrument for measuring digital leadership among clinical nurses. It offers a practical tool for educators, administrators, and researchers to assess and enhance digital leadership capabilities—ultimately supporting the digital transformation of health care systems.

## Introduction

The digital transformation era has accelerated the adoption of digital technologies in the health care sector, particularly after COVID-19, which catalyzed the integration of such technologies into clinical practice [1-3]. Clinical nurses are increasingly using digital tools to ensure safe care environments and to provide evidence-based nursing [4,5]. Common technologies used by nurses include wearable devices, electronic health records, and Barcode Medication Administration systems [6,7], all of which enhance patient safety and efficiency. The adoption of these digital tools in clinical settings has been shown to improve nursing efficiency and patient outcomes [8].

As health care environments become increasingly digitalized, nurses are expected not only to use digital technologies in practice but also to adapt to and support digital change in clinical settings. Nurses serve as coordinators, ensuring effective communication among patients, families, health care professionals, and nursing peers, which is directly related to patient safety [4,9]. In this context, digital leadership encompasses not only the technical ability to understand and apply digital tools [10] but also the capacity to introduce new communication systems and foster collaboration among team members [11]. Therefore, to successfully implement digitalization and health information technologies within hospital organizations, leadership from nurses who continuously enhance and manage their clinical competencies is essential [12,13]. The level of leadership demonstrated by nurses is closely linked to the quality of nursing services [14].

Digital leadership combines traditional nursing competencies with digital capabilities, thereby improving the quality and efficiency of care delivery [8]. In rapidly evolving health care environments, digital leadership is no longer a personal attribute of individual nurses but a comprehensive competency influencing teams and organizations [15]. Nurses must demonstrate adaptability to digital changes [2], solve problems arising during digital transitions [11,16], and support peer education and motivation [2,17]. Effective digital leadership also includes ethical considerations, such as the protection of patient information in the context of big data usage [18]. Existing tools for measuring digital leadership have primarily been developed for managers or employees in general corporate settings, focusing on behaviors and skills required in digital work environments, including vision sharing and resource support. These tools aim to assess general leadership attributes applicable in digital contexts [19,20]. However, they are limited in that they do not reflect the specific characteristics of nursing practice, which involves unique therapeutic relationships between nurses and patients [21], nor do they account for the evolving nature of contemporary clinical settings due to the introduction of new digital technologies [3]. While digital leadership among nurses—encompassing the ability to adapt to digital transformation and effectively integrate digital technologies with health data—is increasingly essential [2,22,23], there remains a lack of measurement tools that are specifically developed to reflect the unique roles and characteristics of clinical nurses.

The purpose of this study is to develop and validate the Digital Leadership Scale for Clinical Nurses (DLS-CN). This scale is intended to accurately assess the level of digital leadership among clinical nurses and provide a foundation for evaluating and enhancing their leadership capabilities in digital health care environments.

## Methods

### Study Design

This study is a quantitative methodological research conducted to develop and evaluate a measurement tool for assessing digital leadership among clinical nurses. The development of the DLS-CN was carried out in 2 phases, based on the tool development process proposed by DeVellis and Thorpe [24]. In the first phase, preliminary items were developed, followed by content validity testing, cognitive interviews, and a pilot survey. In the second phase, the tool was administered to clinical nurses to examine its reliability and validity.

### Scale Development Process

#### Phase 1: Preliminary Item Development

##### Clarification of the Conceptual Definition

This study was based on the hybrid model of Schwartz-Barcott and Kim [25], applying theoretical, fieldwork, and final analytic phases. Based on a previous study analyzing the concept of digital leadership [15], a literature review was conducted, and concept attributes were integrated to establish a conceptual framework and identify core attributes. Through this process, 6 components of digital leadership were identified: digital problem-solving ability, digital ethical sensitivity, digital mindset, self-growth, digital collaboration, and organizational influence.

##### Item Generation

A total of 104 initial items were generated across 6 components of digital leadership for clinical nurses. Specifically, 21 items measured digital problem-solving ability, 19 items measured digital ethical sensitivity, 16 items measured digital mindset, 9 items measured self-growth, 17 items measured digital collaboration, and 22 items measured organizational influence.

##### Selection of Measurement Format

Since the items were designed to measure the level of agreement with statements regarding digital leadership among clinical nurses, the widely used Likert scale was deemed appropriate [24]. Therefore, a 5-point Likert scale was selected.

##### Expert Content Validity Assessment

To evaluate the content validity of the preliminary items, an expert panel of 9 members was formed: 2 clinical nurses with more than 5 years of experience; 2 nurse unit managers; 2 nursing professors; and 1 expert each in digital health care, leadership, and hospital management. After the researchers explained the concept of digital leadership for clinical nurses, the experts rated the appropriateness of the item content and components using a 4-point Likert scale. For item-level evaluation, the item-level content validity index was considered acceptable if it was 0.78 or higher. To assess overall content validity, the scale-level content validity index was calculated by averaging, with 0.90 or higher judged as acceptable [26]. Two rounds of expert validation were conducted. As a result of the first round, 71 items were selected. After the second round, the number was reduced to 46 items. During this process, sentence order and expressions were revised for better clarity and specificity, especially when abstract or ambiguous wording was identified.

##### Cognitive Interviews

Cognitive interviews were conducted with 5 clinical nurses who met the inclusion criteria of the main study. Each interview lasted 30 to 40 minutes and aimed to understand how participants interpreted and responded to the preliminary items. During the interviews, participants were asked to explain their thought processes while answering each item, including how they understood the wording, whether any terms were unclear, and whether the items were easy or difficult to answer. The interview data were analyzed by reviewing participants’ explanations and feedback on item comprehension, wording, and response processes. Based on these findings, items were revised to improve clarity and reduce potential response errors. When similar or overlapping content was identified, the researchers rechecked whether the same concern was expressed by other interviewees before making revisions. The revised items were then reviewed again with the interviewees to confirm their appropriateness. Some participants questioned the appropriateness of the term “hospital information system,” but after reviewing related tools, literature, and expert texts, the term was retained to encompass systems such as electronic medical records and order communication systems. Regarding terms describing digital collaboration tools or systems, alternatives like “intranet” and “groupware” were suggested; however, “digital collaboration system” was found to be the most understandable and suitable. In response to feedback, the term “real-time” was added to better reflect the advantages of system usage. While some general staff nurses noted difficulty adopting new technologies and resources, replacing terms like “utilize” or “apply” was found to weaken alignment with intended attributes, so the original expressions were maintained. After revising the items, the modified 46 items were confirmed for appropriateness through follow-up review with the interviewees.

##### Pilot Study

To identify issues with the preliminary items, a pilot study was conducted with 30 clinical nurses who met the same criteria as those in the main study. This was conducted via an online survey identical in format to the main study. Respondents rated item comprehensibility on a 5-point scale (1=“very difficult” to 5=“very easy”), yielding an overall average of 4.14 (SD 0.24), indicating that the items were well understood. The time required to complete the survey ranged from 5 to 25 minutes, with an average of 11 (SD 1.84) minutes. Open-ended questions confirmed that item length, font size, and type were appropriate. Based on the pilot results, items with duplicate meanings or unclear wording were revised. Additionally, a new item was added to the organizational influence component, “I can help organizational members experiencing difficulties in digitalized work environments,” to complement the existing item, “I can ask organizational members for help when I face difficulties.” All items were revised through linguistic review to ensure correct spacing, terminology, and order. Ultimately, the preliminary item pool was finalized with 47 items across 6 components.

### Phase 2: Instrument Evaluation—Reliability and Validity Testing

#### Participants

Participants were 446 clinical nurses working at tertiary or general hospitals in South Korea. Eligible participants were nurses who were directly involved in patient care and had at least 1 year of clinical experience. Nurses with less than 1 year of experience were excluded because they were considered to be in the novice stage of acquiring the knowledge and skills necessary for nursing practice [27,28]. Nurses in administrative, educational, or nondirect care roles were also excluded. A minimum sample size of 200 is generally recommended for factor analysis, and for confirmatory factor analysis (CFA), a commonly used rule of thumb is that the sample should be at least 10 times the number of estimated parameters [29,30]. Therefore, the total sample size of 446 was considered adequate. To examine the factor structure and cross-validate the model, the total sample was randomly divided into 2 equal groups, with 223 participants assigned to exploratory factor analysis (EFA) and 223 to CFA. This equal split was used to ensure independent and sufficiently large samples for both analyses.

#### Instruments

To evaluate convergent validity, 2 instruments were selected: the Nursing Informatics Competency Scale [31] and the Self-Leadership Scale [32]. The Nursing Informatics Competency Scale includes 20 items across 5 components: “Basic information and communication technology use” (3 items), “Utilization of nursing information” (5 items), “Professional responsibility and ethics” (5 items), “Use of information and communication technology in nursing” (4 items), and “Attitudes toward nursing informatics” (3 items). Each item is rated on a 4-point Likert scale. The reliability of the original scale was a Cronbach α of 0.91 [31], and in this study, internal consistency reliability was assessed using Cronbach α based on the responses of the study participants, with a Cronbach α coefficient of 0.91, indicating excellent internal consistency. The Self-Leadership Scale consists of 16 items across 4 components: “Collaborative self-dialog strategies” (4 items), “Physical vitality enhancement strategies” (4 items), “Goal-oriented self-training strategies” (4 items), and “Self-respect pursuit strategies” (4 items). This instrument uses a 5-point Likert scale. The original reliability was a Cronbach α of 0.86 [32], and in this study, the Cronbach α was also calculated using the current dataset, yielding a coefficient of 0.91, which indicated excellent internal consistency.

#### Data Collection

Data were collected from March 10, 2024, to March 30, 2024, through a self-administered online survey using a nonprobability convenience sampling method. Participants were able to complete the survey at their preferred time and location without direct contact with the researchers. To recruit nurses from diverse regions, recruitment notices containing the contact information of the researchers and the survey link (URL) or QR code were posted on online nursing community boards and workplace forums, allowing eligible participants to voluntarily access and complete the survey. All items were set as mandatory, preventing participants from proceeding without responding; consequently, no missing data were observed in the submitted questionnaires. Data collection continued until the predetermined sample size required for item analysis was reached, after which the survey was closed to prevent additional responses. Because the survey link was distributed through open online communities and workplace forums, the total number of individuals who viewed the recruitment notice could not be determined, and thus the response rate could not be calculated.

#### Data Analysis

Collected data were analyzed using IBM SPSS Statistics version 25.0 and AMOS version 23.0. First, participants’ general characteristics were analyzed using frequencies, percentages, means, and SDs. Second, for the item analysis, means, SDs, skewness, kurtosis, inter-item correlations, item-total correlations, and reliability (if an item was deleted) were calculated. Third, to test the suitability of EFA, the Kaiser-Meyer-Olkin (KMO) measure and the Bartlett test of sphericity were used. Fourth, principal component analysis with varimax rotation was performed to extract factors. Fifth, CFA was conducted to evaluate model fit and test convergent and discriminant validity. Sixth, convergent validity was also examined using the Pearson correlation coefficient. Finally, internal consistency reliability was tested using Cronbach α.

### Ethical Considerations

This study was approved by the Institutional Review Board of Daegu Catholic Medical Center (CR-23-148). The online survey introduction clearly explained the study’s purpose, informed consent, procedures, voluntary participation, confidentiality, potential benefits and risks, and stated that the data would be used only for research purposes. Participants received a small token of appreciation for their involvement. The study was conducted in accordance with the ethical principles of the Declaration of Helsinki.

## Results

### General Characteristics of Participants

A total of 446 clinical nurses participated in the survey (Table 1). Among them, 223 participants were included in the EFA, with an average age of 30.71 (SD 5.45) years. Most participants were female (208, 93.3%). Regarding the type of hospital where they worked, 137 participants (61.4%) were employed at tertiary hospitals and 86 (38.6%) at general hospitals. The most common region was Seoul, the capital, with 106 participants (47.5%). The average length of clinical experience was 70.41 (SD 46.68) months. In terms of work departments, 137 participants (61.4%) worked in general wards, 52 (23.3%) in special units (eg, intensive care units, operating rooms, emergency rooms), 17 (7.6%) in outpatient clinics, and 17 (7.6%) in other departments. For the CFA, 223 participants were included, with an average age of 31.07 (SD 4.81) years. The majority were female participants (200, 89.7%). A total of 131 participants (58.7%) worked in tertiary hospitals and 92 (41.3%) in general hospitals. The highest number of participants was also from Seoul (99, 44.4%). The average length of clinical experience was 74.31 (SD 57) months. Regarding work departments, 147 participants (65.9%) worked in general wards, 38 (17.0%) in special units, 25 (11.2%) in outpatient clinics, and 13 (5.8%) in other departments.

**Table 1.** General characteristics of participants for factor analysis (N=446).

Characteristics and categories		EFA^a^ (n=223)	CFA^b^ (n=223)
Age (y), mean (SD)		30.71 (5.45)	31.07 (4.81)
Sex, n (%)			
Female		208 (93.3)	200 (89.7)
Male		15 (6.7)	23 (10.3)
Marital status, n (%)			
Married		161 (72.2)	163 (73.1)
Unmarried		62 (27.8)	60 (26.9)
Educational level, n (%)			
Associate’s degree		209 (93.8)	206 (92.4)
Bachelor’s degree		13 (5.8)	17 (7.6)
Master’s degree		1 (0.4)	0 (0.0)
Hospital type, n (%)			
Tertiary hospital		137 (61.4)	131 (58.7)
General hospital		86 (38.6)	92 (41.3)
Hospital location, n (%)			
Capital		106 (47.5)	99 (44.4)
Metropolitan		78 (35.0)	82 (36.8)
Province		39 (17.5)	42 (18.8)
Clinical career (y), mean (SD)		5.87 (3.89)	6.19 (4.75)
Working unit, n (%)			
General ward		137 (61.4)	147 (65.9)
Special department (ICU^c^, OR^d^, and ER^e^)		52 (23.3)	38 (17.0)
OPD^f^		17 (7.6)	25 (11.2)
Other		17 (7.6)	13 (5.8)
Experience related to digital leadership education, n (%)			
Yes		13 (5.8)	7 (3.1)
No		210 (94.2)	216 (96.9)
Necessity of digital leadership education, mean (SD)		4.00 (0.89)	4.01 (0.82)

a EFA: exploratory factor analysis.

b CFA: confirmatory factor analysis.

c ICU: intensive care unit.

d OR: operating room.

e ER: emergency room.

f OPD: outpatient department.

### Item Analysis

To examine the distribution of items in the 223 collected responses for factor analysis, the mean, SD, skewness, and kurtosis were calculated for each item. Since none of the items had an absolute skewness value greater than 3.0 or an absolute kurtosis value greater than 8.0, all items met the assumption of normality [33]. Item-total correlation coefficients were reviewed, and items with coefficients below 0.30 or above 0.80 were removed, as these values suggest low contribution to the scale or item redundancy [34]. Seventeen items were removed due to low internal consistency reliability and weak correlation between item pairs. Based on corrected item-total correlation and changes in reliability after item deletion [33], the corrected item-total correlations ranged from 0.43 to 0.72, and the reliability did not significantly change upon item deletion (Table 2). As a result, 30 items were retained for further analysis.

**Table 2.** Goodness-of-fit test for the measurement model (n=223).

Goodness-of-fit	*P* value	Chi-square (*df*)	*χ^²^*/*df^a^*	CFI^b^	RMSEA^c^	SRMR^d^
Measurement model	<.001	661.64 (371)	1.78	0.91	0.06	0.5
Criteria	<.05	—^e^	<3	≥0.90	≤0.06	≤0.08

a *χ*²/*df*: normed chi-square.

b CFI: comparative fit index.

c RMSEA: root mean square error of approximation.

d SRMR: standardized root mean squared residual.

e Not available.

### Exploratory Factor Analysis

EFA using principal component analysis with varimax rotation was conducted on the 30 items of the DLS-CN. Prior to the factor analysis, data adequacy was assessed using the KMO measure and the Bartlett test of sphericity. The KMO value was 0.96, indicating excellent sampling adequacy, and the Bartlett test of sphericity was statistically significant [35], confirming the data’s suitability for factor analysis. To determine the number of factors, eigenvalues greater than 1.0 and a cumulative variance explanation rate exceeding 60% were used based on Kaiser rule. Scree plot analysis and the interpretability of the factor structure were also considered. Items were retained based on communalities greater than 0.40, factor loadings above 0.40, and at least 3 items per factor [36]. One item with a factor loading below 0.40 was removed. The final analysis included 29 items, with a KMO of 0.95 and a statistically significant Bartlett test (*χ*²_820_=4799.3; *P*<.001), indicating the appropriateness of factor analysis. The EFA extracted 4 factors with eigenvalues greater than 1.0, explaining a cumulative variance of 56.9%. Communality values ranged from 0.52 to 0.64, and all factor loadings exceeded 0.40, indicating good model fit. The purpose of factor analysis is to understand the pattern of data and clarify the interpretability of factors [37]. The factor loadings and item contents were examined to ensure conceptual coherence. Cross-loading items that were theoretically essential were assigned to conceptually valid factors. The inter-item correlation coefficients within subfactors were as follows: factor 1: 0.43 to 0.64; factor 2: 0.35 to 0.57; factor 3: 0.35 to 0.51; and factor 4: 0.39 to 0.052, indicating that the model estimation was appropriate (Table 3).

**Table 3.** Communality and pattern matrix of the final exploratory factor analysis (n=223)^a^.

Item number	Communality	Factor loading			
		F1^b^	F2^c^	F3^d^	F4^e^
1	0.58	0.63	0.29	0.30	0.12
46	0.64	0.63	0.05	0.25	0.42
39	0.60	0.62	0.17	0.30	0.31
8	0.61	0.61	0.44	0.16	0.12
9	0.54	0.60	0.32	0.21	0.17
2	0.62	0.60	0.34	0.35	0.18
40	0.63	0.59	0.14	0.24	0.45
45	0.52	0.52	0.33	0.25	0.25
4	0.57	0.49	0.43	0.33	0.21
22	0.66	0.24	0.68	0.20	0.31
14	0.60	0.29	0.68	0.21	0.13
16	0.60	0.31	0.66	0.13	0.24
26	0.58	0.16	0.65	0.29	0.22
30	0.52	0.10	0.55	0.33	0.31
36	0.52	0.30	0.48	0.45	0.06
31	0.62	0.03	0.29	0.62	0.39
33	0.53	0.31	0.20	0.61	0.17
37	0.53	0.40	0.11	0.58	0.14
34	0.54	0.29	0.22	0.58	0.27
20	0.54	0.32	0.34	0.56	0.05
21	0.56	0.20	0.28	0.54	0.39
25	0.54	0.35	0.25	0.49	0.33
19	0.58	0.15	0.27	0.27	0.64
12	0.56	0.26	0.29	0.15	0.63
41	0.59	0.27	0.06	0.42	0.58
18	0.57	0.22	0.46	0.18	0.53
27	0.53	0.20	0.44	0.18	0.52
42	0.52	0.44	0.12	0.28	0.48
38	0.54	0.44	0.36	0.00	0.47
Eigen value	—^f^	13.16	1.26	1.10	1.01
Variance (%)	—	16.31	14.80	13.23	12.60
Cumulative variance (%)	—	16.31	31.11	44.34	56.94

a Kaiser-Meyer-Olkin (KMO) value is 0.95, and the Bartlett test of sphericity was significant (*χ*²_406_=3492.66; *P*<.001).

b F1: ability to use digital technology.

c F2: digital safety management.

d F3: digital collaboration mindset.

e F4: organizational influence.

f Not applicable.

### Confirmatory Factor Analysis

CFA was conducted to verify whether the 4 factors and 29 items extracted during the EFA appropriately reflected the constructs of digital leadership among clinical nurses. The model’s goodness-of-fit was assessed to confirm the adequacy of the structure. To evaluate the model fit, the chi-square statistic was examined. A model was considered acceptable if the normed chi-square *χ*²/*df* was less than 3, the comparative fit index was above 0.90, the root mean square error of approximation was below 0.06, and the standardized root mean squared residual was below 0.80 [38]. The results of the CFA indicated that the chi-square value was statistically significant, and the normed chi-square *χ*²/*df* was 1.8, satisfying the criterion of being below 3. The comparative fit index was 0.91, exceeding the threshold of 0.90. The root mean square error of approximation was 0.06, which meets the criterion for a good model fit. The standardized root mean squared residual was 0.05, which is below the acceptable cutoff of 0.08, indicating that the model had a generally good fit (Table 2).

To verify the convergent validity of each factor, the standardized factor loading was required to be 0.5 or higher, the significance of the unstandardized regression coefficient (critical ratio) was required to exceed 1.97 (*P*<.05), the construct reliability had to be 0.7 or higher, and the average variance extracted (AVE) needed to be 0.5 or higher [39]. The results of the convergent validity test showed that the critical ratio values of the unstandardized regression coefficients ranged from 7.83 to 10.63, significantly exceeding the threshold [30], confirming that the measured items met the conditions necessary for validity testing. The standardized factor loadings for all factors met the criterion of 0.5 or higher. The AVE values for each factor ranged from 0.52 to 0.57, satisfying the minimum requirement of 0.50. The construct reliability values ranged from 0.85 to 0.90, all exceeding the minimum requirement of 0.70. Therefore, the DLS-CN demonstrated adequate convergent validity in terms of item construction. Through this, it was confirmed that 4 factors consistently measured the construct of “Digital Leadership Scale for Clinical Nurses,” and that the 29 items consistently represented the corresponding factors. To assess discriminant validity, interconstruct correlation matrices were examined (Table 4). Discriminant validity was evaluated by checking whether the AVE of each factor was greater than the squared correlation coefficients between the factors [39]. In some cases, such as the ability to use digital technology–digital collaboration mindset, ability to use digital technology–organizational influence, digital safety management–digital collaboration mindset, and digital collaboration mindset–organizational influence, the squared correlations between factors exceeded the AVE of the individual constructs, indicating that discriminant validity was only partially satisfied.

**Table 4.** Correlations between variables and verification of construct validity (n=223).

Variables	Squared correlation (*P* value)				AVE^e^	CR^f^
	F1^a^	F2^b^	F3^c^	F4^d^		
F1	1	—^g^	—	—	0.57	0.91
F2	0.49 (<.001)	1	—	—	0.54	0.85
F3	0.66 (<.001)	0.53 (<.001)	1	—	0.52	0.90
F4	0.71 (<.001)	0.51 (<.001)	0.68 (<.001)	1	0.52	0.90

a F1: ability to use digital technology.

b F2: digital safety management.

c F3: digital collaboration mindset.

d F4: organizational influence.

e AVE: average variance extracted.

f CR: construct reliability.

g Not applicable.

### Convergent Validity Testing

To verify convergent validity, the correlations with the Nursing Informatics Competency Measurement Tool [31] and the Self-Leadership Scale [32] were examined. The correlation with the Nursing Informatics Competency Scale was 0.82 (*P*<.001), and the correlation with the Self-Leadership Scale was 0.83 (*P*<.001). Since the correlation coefficients were all above 0.70, convergent validity was confirmed.

### Reliability Testing

Reliability was tested using Cronbach α, with a criterion of 0.70 or higher [29,40]. Cronbach α for the 29 items measuring DLS-CN was 0.95.

By subfactor, Cronbach α was as follows: factor 1 (ability to use digital technology): 0.88; factor 2 (digital safety management): 0.78; factor 3 (digital collaboration mindset): 0.86; and factor 4 (organizational influence): 0.87.

Since Cronbach α for all subfactors and the overall tool exceeded 0.70, reliability was confirmed.

### Final Items

In this study, the final version of the DLS-CN consisted of 29 items across 4 factors (Table 5).

By subfactor, the items were as follows: 8 items for the ability to use digital technology, 5 items for digital safety management, 8 items for a digital collaboration mindset, and 8 items for organizational influence.

**Table 5.** Final items of the convergence of Digital Leadership Scale for Clinical Nurses.

Factors and items		Item number
Ability to use digital technology		
I can provide patient-centered care by using digital technology.		1
I can explore ways to improve digital systems and processes for patient care and treatment.		46
I can explain the importance of actively using digital technology.		39
I can actively use new information obtained through digital technology to solve patients’ health problems.		8
I can present new information obtained through digital technology to address issues in the nursing work environment.		9
I can systematically collect data on patients’ health status and nursing needs using digital technology.		2
I can argue for the necessity of applicable resources (human, systems, equipment, etc) in the digitalizing clinical environment.		45
I understand the principles of digital technology necessary for nursing tasks and can apply them to patients.		4
Digital safety management		
I can learn methods for using and managing new digital technologies applicable to patient care.		22
I can maintain a secure environment for protecting patient privacy in the digitalizing clinical setting (eg, logging out of computers when changing locations).		14
I perform nursing tasks in accordance with organizational guidelines and procedures for managing personal information in the digital clinical environment.		16
I believe self-development is important for improving digital knowledge and technical skills related to nursing tasks.		26
I can request help from organizational members when encountering difficulties in the digital clinical environment.		30
Digital collaboration mindset		
I can assist organizational members who are struggling in the digital clinical environment.		31
I can logically express my opinions so that organizational members can understand them using digital collaboration systems (eg, EMR^a^, PACS^b^, in-hospital messenger, etc).		33
I feel comfortable exchanging real-time opinions through digital collaboration systems (eg, EMR, PACS, in-hospital messenger, etc).		37
I can understand organizational members’ opinions easily when communicating via digital collaboration systems (eg, EMR, PACS, in-hospital messenger, etc).		34
I am not afraid of adapting to digital changes in the clinical setting.		20
If I do not know how to use a new digital technology, I can resolve the issue by using available resources.		21
I can proactively use digital technology to learn the latest nursing knowledge and skills.		25
I can accurately hand over information and nursing processes related to patients using digital collaboration systems (eg, EMR, PACS, in-hospital messenger, etc).		36
Organizational influence		
I am interested in new digital changes that benefit patient care.		19
I reflect on my values and norms that may affect patient privacy in the digital clinical environment.		12
I can encourage organizational members who attempt change by learning new digital technologies.		41
I understand the importance of using new digital technologies for advancing the nursing field.		18
I feel a sense of accomplishment when I effectively perform nursing tasks using newly acquired digital technology.		27
I can help improve organizational members’ digital competencies for better work performance.		42
I strive to achieve organizational goals by introducing new digital technologies and resources.		38
I can present a vision (organizational future image) for performing nursing tasks using digital technology.		40

a EMR: electronic medical record.

b PCA: principal component analysis.

## Discussion

### Principal Findings

This study was conducted to develop a tool to measure digital leadership among clinical nurses, based on the instrument development steps proposed by DeVellis and Thorpe [24]. The research was carried out in 2 phases: the development of preliminary items and the evaluation of the instrument’s validity and reliability.

During the item development phase, a conceptual framework was established by integrating the components of digital leadership identified in previous concept analyses and literature reviews on digital leadership in clinical nursing. Preliminary items were selected from the initial items through a content validity assessment by experts, followed by cognitive interviews and a pilot survey, which were used to derive the final set of preliminary items. Throughout this process, expert feedback and identified issues were systematically reviewed and refined.

In the instrument evaluation phase, data were collected from 446 clinical nurses through an online survey. A random sample of 223 responses was used for EFA, and another 223 responses were used for CFA. Construct validity was tested through these analyses, and convergent validity was assessed by examining the correlation with instruments measuring nursing informatics competency and self-leadership. Internal consistency reliability was confirmed with a Cronbach α of 0.95.

The final tool developed through this study consists of 29 items across 4 factors: 8 items for the ability to use digital technology, 5 items for digital safety management, 8 items for a digital collaboration mindset, and 8 items for organizational influence. The tool uses a 5-point Likert scale for self-report surveys, with a total score range from 29 to 145 points. Higher scores indicate a higher level of digital leadership among clinical nurses. Overall, the developed scale demonstrated strong reliability and validity for assessing digital leadership in clinical nursing practice. However, the tool did not fully satisfy the criteria for discriminant validity. Even when variables are used as conceptually independent constructs, correlations between related factors are common in practice [29]. This is consistent with findings from a study on the development of an integrated nursing leadership scale for Korean nurses [41], which also reported overlaps among constructs such as data management and communication technologies. In particular, relatively high correlations were observed between the ability to use digital technology and organizational influence, as well as between digital collaboration mindset and organizational influence. These results suggest that while the factors are conceptually distinct, they may be closely related in actual clinical settings where digital competencies are integrated with leadership behaviors. Although some aspects of discriminant validity were supported, these findings indicate that the boundaries between certain factors were not clearly distinguished. Despite these limitations, this tool has the advantage of including items that measure competencies needed to adapt to rapidly changing digital health care environments, such as work performance, learning, and adaptability [42]. Accordingly, future research is needed to more clearly distinguish the unique conceptual domains measured by each factor. Furthermore, the “digital safety management” factor included fewer items compared to other factors. Given the importance of patient safety and information security in digital health care environments, future research should consider expanding and refining items in this domain to enhance the comprehensiveness of the scale.

Given that leadership competencies required in digitally transforming health care environments are increasingly complex and diverse [13], this DLS-CN reflects the unique characteristics of Korean clinical settings and is the first scale of its kind to undergo statistical validation. Nevertheless, because data collection was conducted via an online survey using convenience sampling, caution is needed when interpreting the results. Additionally, the scale was developed for general clinical nurses and excluded nurses with less than 1 year of experience, nurse managers not involved in direct care, and those in administrative or educational roles, thereby limiting its generalizability. Moreover, the study did not include test-retest reliability, which represents another limitation.

Based on these findings, several implications can be drawn. In particular, the developed digital leadership scale should be applied to nurses from diverse countries and cultural backgrounds, as well as to nurses in various roles and settings, such as hospitals, community health centers, and educational institutions. Repeated studies are recommended to verify the reliability and validity of the instrument in diverse environments.

Future research should use this tool to assess digital leadership levels and develop tailored educational or intervention programs that enhance digital leadership according to workplace and contextual characteristics.

In addition, as this scale may be applicable not only to health care professionals but also to professionals in management, public administration, and related fields, further research should explore its broader applicability across disciplines.

### Conclusions

The DLS-CN, developed through this study, is a systematic tool designed to measure and evaluate digital leadership among nurses in clinical practice settings. The DLS-CN consists of 4 core domains: ability to use digital technology, digital safety management, digital collaboration mindset, and organizational influence. It proposes a new standard for nursing leadership in health care environments driven by digital technologies.

In particular, the tool is useful for developing various educational programs aimed at identifying emerging nurse leaders and enhancing the digital leadership of clinical nurses in hospital environments where frequent changes in digital health care systems, technologies, and organizational structures occur. Furthermore, the development of this tool confirms that digital leadership extends beyond the mere acquisition of digital skills, encompassing the competence of clinical nurses to actively engage in and lead change. This is a meaningful contribution to the study.

Since this tool was developed based on data collected from clinical nurses in South Korea, and the items were created accordingly, further validation studies are needed in diverse health care settings and across cultural contexts. In the rapidly evolving digital health care environment, this tool can serve both as a foundational resource and a structured instrument for strengthening clinical nurses’ digital leadership.

## Abbreviations

AVE: average variance extracted
CFA: confirmatory factor analysis
DLS-CN: Digital Leadership Scale for Clinical Nurses
EFA: exploratory factor analysis
KMO: Kaiser-Meyer-Olkin
